# Current status of global pig production: an overview and research trends

**DOI:** 10.5713/ab.23.0367

**Published:** 2023-11-02

**Authors:** Sung Woo Kim, Alexa Gormley, Ki Beom Jang, Marcos Elias Duarte

**Affiliations:** 1 Department of Animal Science, North Carolina State University, Raleigh, NC 27695, USA

**Keywords:** Feed Additives, Intestinal Health, Nutrition, Pig Production, Pork Production, Research Trends

## Abstract

Global pig production has increased by 140% since the 1960s. The increase in global population, coupled with improving socioeconomic conditions of many countries has led to an increased consumption of meat globally, including pork. To keep up with demand and capitalize on economic opportunities, the countries of China, the United States (US), and the European Union (EU) have become the top 3 pork producers globally. China is of particular interest, as it is the both the largest country in pork production and pig numbers, as well as being the largest importer of pork from other countries. Globally, the efficiency of pork production has improved, in relation to the integration of pig production and the dramatic increase in research efforts in pig nutrition and production. Through integration, large producers can consolidate resources and maximize profits and efficiency. The increased research interest and efforts in pig production have given scientists and producers the opportunity to collaborate to adapt to challenges and identify possible solutions to issues brought on by a volatile global market. Intestinal health (23%), general nutrition and growth (23%), and amino acid nutrition (15%) were the top 3 areas (61%) leading research trends in pig nutrition and production. Major dietary interventions with feed additives evaluated include functional amino acids, feed enzymes, pre-/pro-/post-biotics, and phytobiotics with a common goal to improve the growth efficiency by enhancing nutrient utilization and intestinal health. With increasing global issues with environment, pig producers and the supporting scientists should continue their efforts to improve the production efficiency and to reduce the environmental footprint from pig production.

## INTRODUCTION

**Figure 1:**
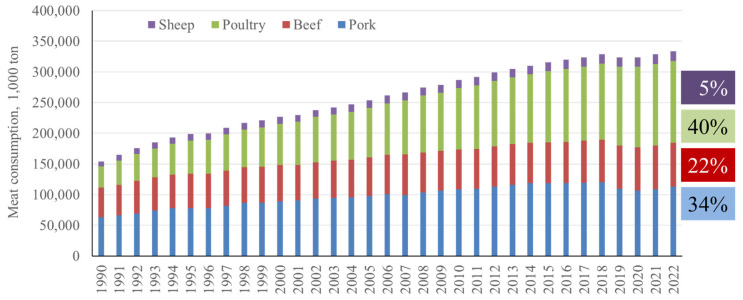
Consumption of various meats from 1990 to 2022 [1].

**Figure 2:**
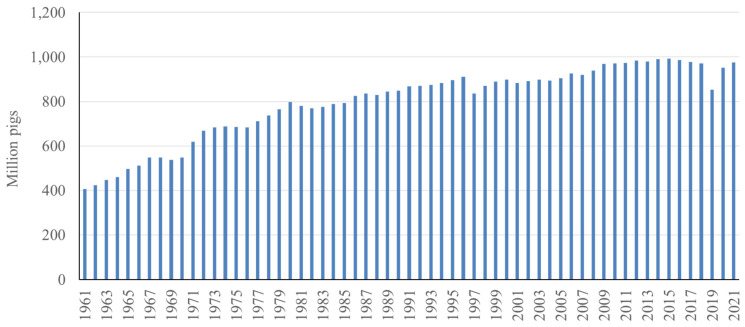
Number of pigs raised in the world from 1961 to 2021 [2].

Pork is one of the most consumed meats globally. A recent survey indicates that in 2022, 34% of the meat consumed globally was pork, whereas poultry and beef took 40% and 22%, respectively [1] (Figure 1). Global pork consumption has increased by 77% from 63.5 million tons in 1990 to 113 million tons in 2022, whereas poultry and beef saw a 287% and 49% increase during the same period, respectively. With the growing global population and economic development, this trend in increasing pork consumption will continue, requiring an increased supply of pork. Pork is derived from pig production and there are almost 1 billion pigs raised globally [2]. Similar to the continuous increase of pork consumption, global pig production has also increased by 140% from 1961 to 2021 [2] (Figure 2). In this article, changes in pig numbers in different continents and variation in production efficiency globally are reviewed in further detail.

**Figure 3:**
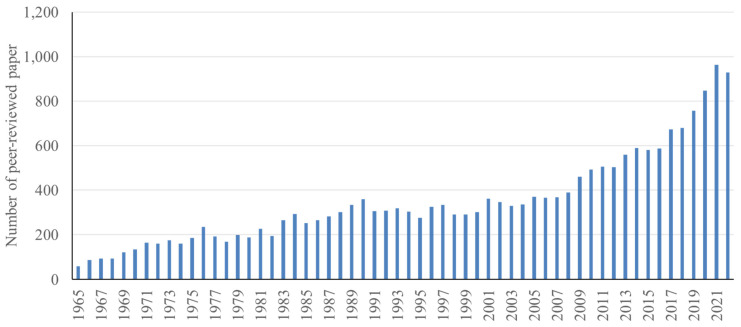
The number of peer-reviewed papers published in scientific journals in the area of pig production, pig nutrition, and pig growth from 1965 to 2022 [4].

Along with increases of pork consumption and pig numbers, the efficiency of pig production has also improved. Advances in research have enabled this improvement. Based on the database provided by National Center for Biotechnology Information (NCBI), the number of published papers in pig production including nutrition and growth physiology has been dramatically increased from 60 papers in 1965 to nearly 1,000 papers in 2022 [3,4] (Figure 3). Thus, the recent trends and issues in pig production research are also reviewed in this article. Finally, based on the review of changes to various aspects of pig production from the past to present, contemporary issues in global and regional pig production are discussed in this article.

## GLOBAL PIG PRODUCTION AT A GLANCE

**Figure 4:**
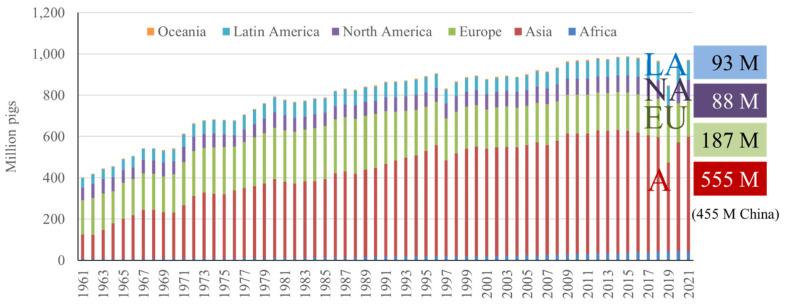
Number of pigs in different continents from 1961 to 2021 [2]. LA, Latin America; NA, North America; EU, Europe; A, Asia.

**Figure 5:**
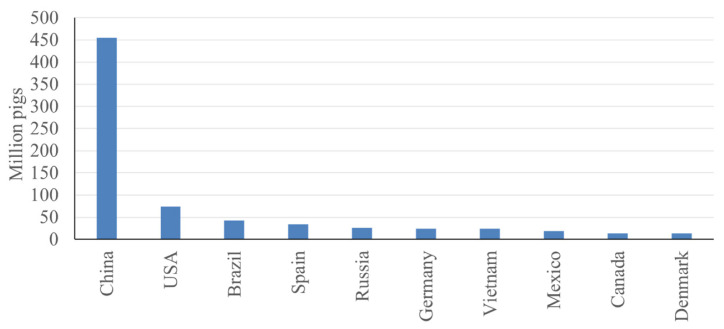
Top 10 countries in pig number, estimated in 2021 [2].

Currently, Asia has the greatest number of pigs (57%), followed by Europe (19%), Latin America (9.6%), North America (9.1%), and Africa (4.4%) [2] (Figure 4). This remains consistent with previous trends in that Asia has maintained the greatest number of pigs per continent since the 1960s [2] (Figure 4). The high pig population in Asia can primarily be attributed to China, the country with the greatest number of pigs in the world. When looking at the top 10 countries in terms of number of pigs, China has a significant lead over the next closest countries, with approximately 450 million pigs, and the second largest individual country, the United States (US), having less than 80 million pigs [2] (Figure 5).

**Figure 6:**
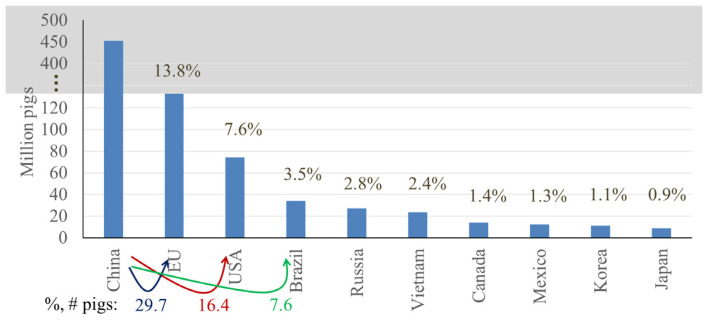
Top 10 countries in pig number, estimated in 2023 [5,6]. %, # pigs indicates the relative number of pigs produced in comparison to China.

**Figure 7:**
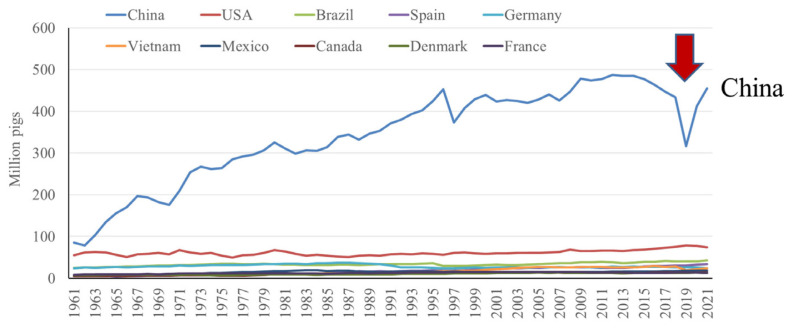
Top 10 countries in pig number by year, emphasis on China [2].

These top 10 countries contain 81% of the pigs globally, with the top 3 countries, China, the European Union (EU: considering the EU as one entity for the purpose of this communication), and the United States, possessing 46.4%, 13.8%, and 7.6% of the global pig population, respectively [5] (Figure 6). When considering the next 3 highest ranking countries, the EU has only 29.7% the number of pigs found in China, whereas the US and Brazil have even less, at 16.4% and 7.6% the number of pigs found in China, respectively. Considering China, the total number of pigs has been steadily increasing since 1960, with a marked decline in population beginning in 2015 due to the effects of African Swine Fever (ASF), however, since then, the pig population in China has been recovering [2] (Figure 7).

**Figure 8:**
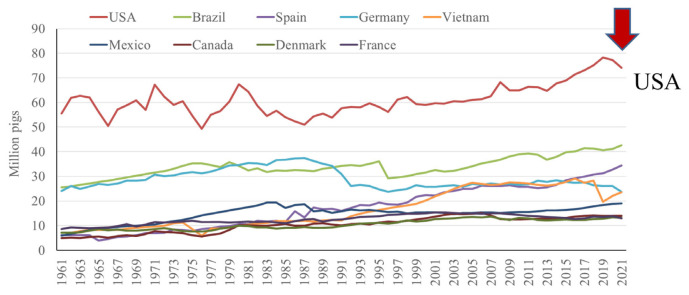
Top 10 countries in pig number by year, excluding China [2].

**Figure 9:**
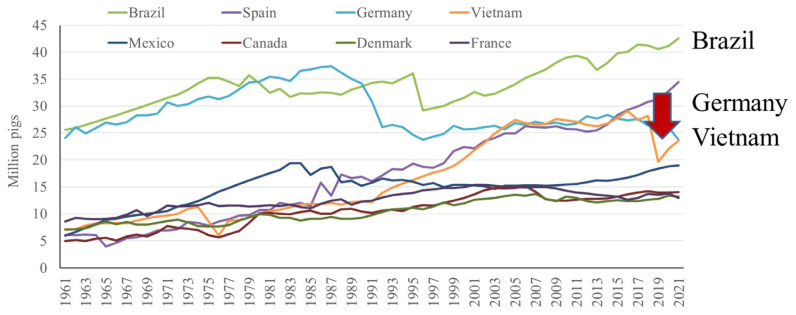
Top 10 countries in pig number by year, excluding China and the US [2].

In contrast, the US pig population has seen a less dramatic increase in pig population, with a steady increase from 1980s to 2019, where then there is a decrease in pig numbers in response to large integrators reducing the population of their herds [2] (Figure 8). In general, a gradual increase in pig numbers has been observed in all of the top performing countries over the last sixty years, with the exception of Germany, where numbers have been decreasing since approximately 2014, and Vietnam, where ASF led to a sharp decline in population around 2018, from which they are still recovering [2] (Figure 9).

## PRODUCTION EFFICIENCY

**Figure 10:**
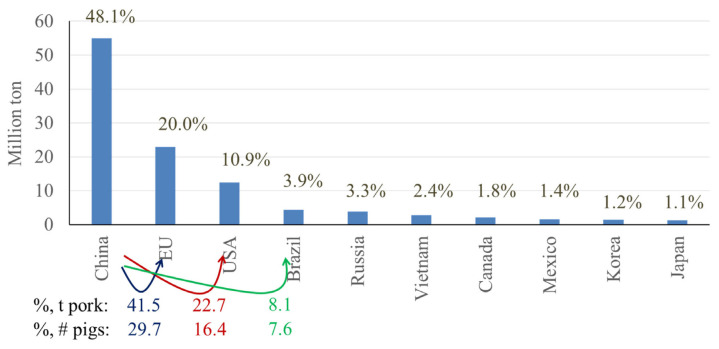
Top 10 countries in pork production in 2023, in million ton [2,5,7]. %, t pork indicates the relative amount (%) of pork produced in comparison to China; %, # pigs indicates the relative number of pigs produced in comparison to China.

In addition to number of pigs, the top producing countries can also be quantified by their pork production. The top 10 countries in terms of number of pigs made up 81% of the global pig population, however, these same countries are estimated to produce 94% of all pork globally [5–7] (Figure 10). Interestingly, the proportion of pork produced by each country is not the same as the proportion of total pigs in these countries when both are compared relative to China. This difference can be attributed to variation in production efficiency and other factors among countries. Production efficiency can include feed efficiency, lean gain potential, mortality, and morbidity. Other factors can include market weight and trading of live animals.

Statistics based on data from Pig Progress [5] and USDA [6,8] are shown in Figure 10 and Table 1 illustrating that when compared to China, the EU, US, and Brazil have 29.7%, 16.4%, and 7.6% the number of pigs, respectively, whereas these same countries produced 41.5%, 22.7%, and 8.1% the amount of pork, respectively. In terms of efficiency, the EU produces 40% and the US produces 38% more pork per the same number of pigs when compared with China. Some of this variation in efficiency can be explained by a difference in market weight between China and other countries, as China tends to market pigs at approximately 105 kilograms (kg) bodyweight whereas the EU and the US market pigs at approximately 130 kg bodyweight. In addition to market weight differences, there may be some variation in growth efficiency due to additional factors such as feed composition, genetics, management, mortality, morbidity, and environment.

## PORK CONSUMPTION PATTERN AND PORK TRADING

Based on data of pork production from USDA [7] and USDA FAS [6,8] and human population data from the United Nations [9] and The World Bank [10], pork consumption per capita was calculated considering total pork production, the amount of pork imported, and the amount of pork exported to calculate the domestic pork consumption. The domestic pork consumption was divided by the human population in Table 2.

Despite their overall consumption being incredibly high, China ranks number 3 in annual pork consumption per capita at 39.9 kg/capita, falling behind the EU and Korea, with these countries consuming an average 42.7 and 41.5 kg/capita, respectively [6–10] (Table 2). For example, it can be estimated that 66% of all meat consumed in China is pork, whereas other large pork producing countries consume less pork compared with China, with the EU, US, Canada and Brazil consuming pork as 63%, 24%, and 19% of their total meat consumption, respectively. Interestingly, China ranks relatively low in terms of total meat consumption per person (61 kg/capita) annually, whereas other countries have a relatively higher total annual meat consumption per person, like the US (124 kg/capita), Canada (83 kg/capita), and Brazil (77 kg/capita) (Table 2). Despite this, pork consumption and production in China is still expected to increase as consumer demand remains high. It is also interesting to observe that pork was 66% of total meat consumed in China, 63% in the EU, and 59% in Korea, whereas it was 19% in Brazil, 24% in the USA, and 31% in both Canada and Mexico. High preference of pork to other meats in China, the EU, and Korea are related to cultural and historical backgrounds. In the case of Brazil, the preference of consuming pork was low due to high beef consumption, whereas the USA, Canada, and Mexico had high poultry meat consumption.

**Figure 11:**
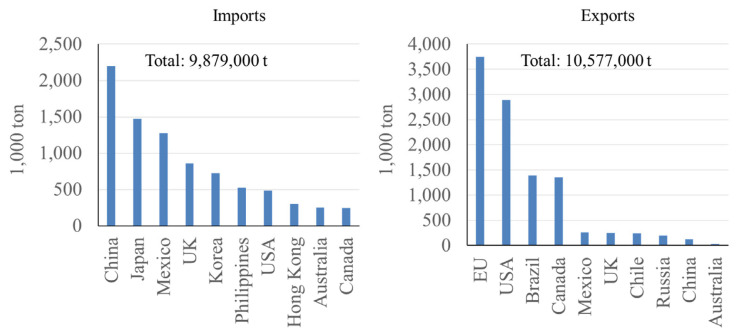
Top 10 countries for pork imports and exports, estimated in 2023, in thousand ton [8].

In 2023 it was estimated that among the top 10 pork trading countries, 9.9 million tons of pork were imported and 10.6 million tons of pork were exported between these countries [8] (Figure 11). Despite being the largest pork producer globally, China imports more pork than any other country, followed by Japan and Mexico. It is interesting to note that although China produces over twice the amount of pork compared to any other country, China still imports the greatest amount of pork of any country at 2.5 million tons (Table 2). This can be attributed to both the large population of China and cultural differences leading to China consuming pork in higher proportions when compared with other countries. In contrast, the next highest ranking pork producing countries, the EU and the US, rank first and second in pork exports, respectively [8] (Figure 11).

## GLOBAL TREND OF INTEGRATION IN PIG PRODUCTION

**Figure 12:**
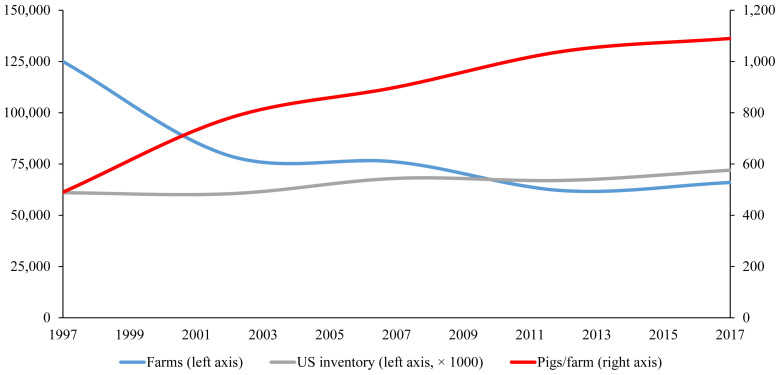
Trends in the number of pig farms, pig inventory, and pigs per farm in the United States (1997–2017) [7].

As pig production expands globally, pig producers and feed mills turn to integration to increase efficiency and maximize profits [11,12]. For example, the US has seen a drastic change in how large pig producers raise animals over the last 25 years (Figure 12) [7]. Table 3 illustrates that in 1995, the top 25 pig producers in the US owned approximately 1.3 million sows, whereas in 2020, the top 25 pig producers owned approximately 4.1 million sows, 65% of the total sow population in the US [13]. Prolific pig producers in major pig producing countries acquire other smaller companies and independent producers in an effort to integrate all aspects of production. As previously mentioned, integration can improve profits and production efficiency, which allows for greater access to and consolidation of resources, creating a cyclical pattern that encourages systemic integration within the industry.

Similar to the US swine industry, global pig production has become integrated, leading to mega-producers, providing new opportunities, and also new challenges. Globally, the top 10 pig producers own 11 million sows, or 17% of the world’s total sow population (Table 4). This can be compared to the top 40 global pig producers that own 26% of the world’s total sow population, meaning that the next 30 largest global pig producers own only 9% of sows worldwide, a large difference when compared with the striking 17% contributed by the top 10.

The top 10 global pig producers are found in the countries of China (6), the US (2), Thailand (1), and Brazil (1) (Table 4) [14,15]. Massive pig producers have the ability to support all aspects of pig production, including manufacturing their own feed, hiring nutritionists and veterinarians, and utilizing company-specific harvest facilities. By consolidating all aspects of production, pig producers can decrease internal input costs and maximize profits. This large-scale integration can also contribute to decreased environmental impacts as many of these processes can be streamlined to minimize waste. For example, a large company may choose to build a new feed mill close to their large sow farm to minimize the environmental impact associated with transporting large quantities of feed. This could also minimize the risk of disease transmission that could occur during transport.

## PIG PRODUCTION AND ADVANCES IN RESEARCH

**Figure 13:**
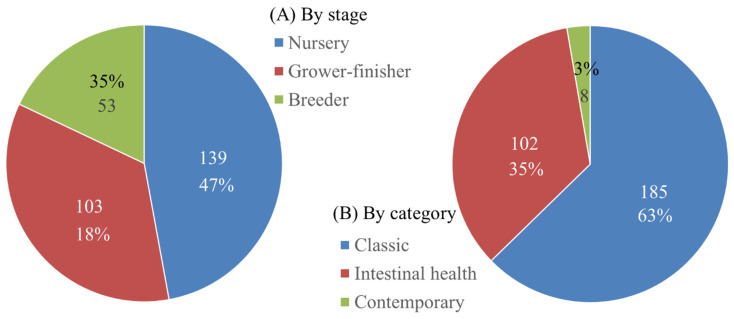
The number of research presentations made at the American Society of Animal Science Annual conference (Albuquerque, NM, USA) and Midwest section conference (Madison, WI, USA) in 2023. (A) By the stage of growth and physiological status, and (B) by major research subject matter.

With the increase in pig production, there has been a simultaneous increase in research efforts in the areas of pig nutrition and production. Kim and Duarte [3] demonstrated that the number of research papers in the area of pig nutrition has increased by 11-fold, from 90 in 1975 to 1,000 in 2020. These research topics provide a good reflection of the current issues, challenges, and trends, as research funding is tightly related to the present goals of the swine industry. Recent research topics were reviewed for the purpose of identifying trends in pig nutrition and production. Two major international conferences held in 2023 were selected: the American Society of Animal Science (ASAS) Annual conference (Albuquerque, NM, USA; July 2023) and Midwest section conference (Madison, WI, USA; March 2023). The science programs of these conferences were reviewed to select research presentations made in the area of pig nutrition and production. These international conferences include participants from all major parts of the world and have been leading conferences where new findings are reported and discussed. The contents of all presentations were checked and grouped in different categories: (A) by the stage of growth and physiological status and (B) by major research subject matter (Figure 13).

There were 295 presentations made at these two conferences. Among the 295 presentations, 47% targeted nursery pigs, 35% targeted grower-finisher pigs, and 18% targeted breeding pigs. Pig nutrition and production research largely focuses on nursery pigs. This is due to the complications associated with weaning, as nursery pigs are weaned at an early age when the gastrointestinal tract is not fully mature and able to handle the typical feedstuffs provided to them [16–18]. When the presentations were grouped by the major research subject matter, research related to classical nutrition and productivity took the largest portion (63%, 185 presentations). However, research subjects related to intestinal health also made up a significant portion of the presentations (35%, 102 presentations), clearly demonstrating the importance of this subject matter in pig production, as the increase in the occurrence of enteric diseases and post-weaning diarrhea have recently been a major concern in the area of pig production [19].

**Figure 14:**
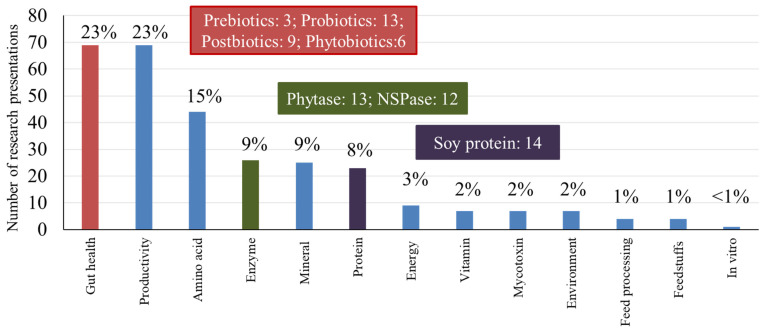
The number of research presentations in the American Society of Animal Science Annual conference (Albuquerque, NM, USA) and Midwest section conference (Madison, WI, USA) in 2023 by 13 research subject matters.

The research subject matters were further categorized to 13 areas including amino acids, energy, environment and smart farming, feed enzymes, feed processing, feedstuffs, growth and productivity, *in vitro*, intestinal health, minerals, mycotoxins, protein supplements, and vitamins. Figure 14 shows the number of presentations made in each category. Research on general growth and productivity made up 23% of all presentations, whereas another 23% of the presentations were focused on intestinal health. These together make up almost half of all research presentations made in the area of pig nutrition and production in 2023. Research on amino acids (15%), feed enzymes (9%), minerals (9%), and protein supplements (9%) took another significant portion of research presentations. Other areas of research include energy feeds (3%), vitamins (2%), and mycotoxins (2%). It is worth noting that 7 presentations (2%) were related to the environment and smart farming. Within the 69 presentations from the ‘intestinal health’ category, 31 presentations were related to prebiotics, probiotics, postbiotics, and phytobiotics, evaluating their impacts on intestinal microbiota and health, often with the use of bacterial or other external challenges. Forty-four presentations (15%) were associated with the use of supplemental amino acids in relation to their requirements, but also the functional role of specific amino acids. Research on feed enzymes for their evaluation and application is another emerging area in pig nutrition and production with 26 presentations at these two conferences. These presentations were mostly about the use of phytase and xylanase (enzymes targeting xylans and arabinoxylans) for the release of phosphorus from phytic acid and xylose oligosaccharides, and their impacts on intestinal health. It was also interesting to see 14 presentations related to the quality of soybean meal and the impacts of soy protein on the intestine of nursery pigs.

From the review of research in the area of pig nutrition and production, it is clear that intestinal health is regarded as an important topic. With the ban of the use of anti-bacterial growth promotors and high-level zinc oxide in many countries globally, research targeting intestinal health and dietary interventions to mitigate intestinal challenge are of high priority in the areas of pig nutrition and production. Major dietary interventions evaluated include functional amino acids, feed enzymes, pre-/pro-/post-biotics, and phytobiotics. The importance of these feed supplements cannot be understated, and thus, the attention received at conferences is justified. The collective goal of these research efforts is to improve the growth efficiency by enhancing nutrient utilization and intestinal health. This is critically important in pig production for sustainable and circular agriculture in an effort to reduce the environmental footprint whilst increasing the production of valuable pork.

## CONCLUSION

Pig production supports 34% of global meat consumption and the needs of pork production continuously increase. Improving the efficiency of pork production is the most critical goal for the pig producers and supporting scientists. Production efficiency varied among major pig producing countries due to differences in market weight, feed composition, genetics, management, mortality, morbidity, and environment. Globally, the efficiency of pork production has improved, in relation to the integration of pig production and the dramatic increase in research efforts in pig nutrition and production. There have been tremendous research efforts in pig nutrition and production primarily focused on intestinal health and growth, both of which target the efficiency improvement. With increasing global issues with environment, pig producers and the supporting scientists should continue their efforts to improve the production efficiency and to reduce the environmental footprint from pig production.

## Figures and Tables

**Table 1:** Ratios of pork production to pig numbers (pork/pig) of top 10 countries relative to China

Country	Pork (million t)	Pig million	Relative to China		Pork/pig

			Pork	Pig	
China	55.0	452.6	100.0	100.0	1.00
EU	22.9	134.3	41.5	29.7	1.40
USA	12.5	74.4	22.7	16.4	1.38
Brazil	4.4	34.3	8.1	7.6	1.07
Russia	3.8	27.1	6.9	6.0	1.15
Vietnam	2.8	23.5	5.0	5.2	0.96
Canada	2.1	13.9	3.8	3.1	1.22
Mexico	1.6	12.3	2.9	2.7	1.08
Korea	1.4	11.1	2.6	2.5	1.04
Japan	1.3	8.9	2.4	2.0	1.20

Pig Progress [5]; USDA [6,8].

**Table 2:** Trends in pork production, import, export, and domestic pork consumption of top 10 pork producing countries

Country	P	I	E	C	Pop	C/Pop	I/Pop	MtC	C/MtC
	
	Million ton	Million ton	Million ton	Million ton	Million ton	kg/ca	kg/ca	kg/ca	%
China	55.0	2.5	0.1	57.4	1439.3	39.9	0.7	61	66
EU	22.9	-	3.8	91.1	446.8	42.7	0.0	68	63
USA	12.5	0.5	2.9	10.1	331.0	30.4	1.5	124	24
Brazil	4.4	-	1.4	3.1	212.6	14.36	0.0	77	32
Russia	3.8	-	0.2	3.6	145.9	24.7	0.0	77	32
Vietnam	2.8	0.2	-	2.9	97.3	30.0	1.7	63	47
Canada	2.1	0.3	1.4	1.0	37.7	25.3	6.5	83	31
Mexico	1.6	1.3	0.3	2.6	128.9	20.3	9.9	65	31
Korea	1.4	0.7	-	2.1	51.3	41.5	14.1	71	59
Japan	1.3	1.5	-	2.8	126.5	21.9	11.6	49	44

P, pork production (million ton); I, pork import (million ton); E, pork export (million ton); C, domestic pork consumption (million ton); Pop, human population; C/Pop, domestic pork consumption/human population; I/Pop, pork import/human population; MtC, total meat consumption; C/MtC, domestic pork consumption/total meat consumption.

USDA [6–8]; The United Nations [9]; The World Bank [10].

**Table 3:** Number of sows (in 1,000 sows) in the top 10 largest companies in the US, including top 25 companies as a percentage of all sows in the US

Rank	Pork producer (×1,000)	1995	2000	2005	2010	2015	2020
1	Smithfield Food	461	695	798	877	894	930
2	Seaboard Foods	50	175	214	214	217	345
3	Pipestone System	-	53	110	140	170	282
4	Iowa Select Farms	42	96	150	158	165	242
5	The Maschhoffs	-	-	115	137	218	195
6	Prestage Farms	96	122	140	125	170	185
7	JBS	80	110	94	119	175	167
8	Carthage System	-	-	-	85	120	160
9	Christensen Farms	-	74	149	163	170	148
10	AMVC Management	-	-	65	77	113	145
	Top 25 Companies	1,316	2,343	2,573	2,918	3,431	4,074^*^

* 65% of total.

Davis et al [13].

**Table 4:** Top 40 pork producing companies globally, ranked by number of sows in 2020

Rank	Pork producer	# sows
1	Muyuan Foodstuff (China)	2,624,000
2	Wens Group (China)	1,800,000
3	Smithfield Foods (USA)	1,225,000
4	Zhengbang Group (China)	1,200,000
5	New Hope Group (China)	1,200,000
6	CP Foods (Thailand)	1,180,000
7	Techbank Food Co., Ltd (China)	500,000
8	Triumph Foods (USA)	443,200
9	Sichuan Dekon Group (China)	400,000
10	BRF S.A. (Brazil)	388,500
Global top 10 companies (17%)		10,960,700
11	Pipestone System (USA)	384,000
12	Seaboard Foods (USA)	340,000
13	Twins Group (China)	250,000
14	Yangxiang (China)	250,000
15	Cooperl (France)	245,000
16	Iowa Select Farms (USA)	242,500
17	DaBeiNong (China)	230,000
18	COFCO (China)	220,000
19	Vall Companys Group (Spain)	213,000
20	Seara Foods (Brazil)	213,000
21	Aonong Group (China)	200,000
22	The Maschhoffs (USA)	187,000
23	Miratorg (Russia)	180,000
24	Aurora (Brazil)	180,000
25	Prestage Farms (USA)	178,000
26	JBS (USA)	169,000
27	Carthage System (USA)	165,600
28	Jiahe Agricultural (China)	160,000
29	AMVC Manag. Services (USA)	152,000
30	Costa Food Group (Spain)	150,000
31	Agrosuper (Chile)	140,000
32	Tecon (China)	135,000
33	Olymel (Canada)	134,000
34	HyLife Ltd./Charoen (Canada)	132,000
35	Betagro (Thailand)	130,000
36	Rusagro (Russia)	125,000
37	Frimesa Coop (Brazil)	120,000
38	Clemens Food Group (USA)	111,000
39	Elephant Agriculture (China)	100,000
40	TRS Group (China)	100,000
	Global top 40 companies (26%)	16,496,800

Feed Strategy [14]; Zhang et al [15].
