# CORRIGENDUM

**DOI:** 10.5713/ab.23.0367C

**Published:** 2025-02-25

The followings are to be changed in **Anim Biosci 2024 Vol 37, No. 4 : 719–729.**

The amount of domestic pork consumption (C) in EU in Table 2 should be 19.1 (not 91.1).

**Table 2:** Trends in pork production, import, export, and domestic pork consumption of top 10 pork producing countries

Country	P	I	E	C	Pop	C/Pop	I/Pop	MtC	C/MtC
	
	Million ton	Million ton	Million ton	Million ton	Million ton	kg/ca	kg/ca	kg/ca	%
China	55.0	2.5	0.1	57.4	1439.3	39.9	0.7	61	66
EU	22.9	-	3.8	**19.1**	446.8	42.7	0.0	68	63
USA	12.5	0.5	2.9	10.1	331.0	30.4	1.5	124	24
Brazil	4.4	-	1.4	3.1	212.6	14.36	0.0	77	32
Russia	3.8	-	0.2	3.6	145.9	24.7	0.0	77	32
Vietnam	2.8	0.2	-	2.9	97.3	30.0	1.7	63	47
Canada	2.1	0.3	1.4	1.0	37.7	25.3	6.5	83	31
Mexico	1.6	1.3	0.3	2.6	128.9	20.3	9.9	65	31
Korea	1.4	0.7	-	2.1	51.3	41.5	14.1	71	59
Japan	1.3	1.5	-	2.8	126.5	21.9	11.6	49	44

P, pork production (million ton); I, pork import (million ton); E, pork export (million ton); C, domestic pork consumption (million ton); Pop, human population; C/Pop, domestic pork consumption/human population; I/Pop, pork import/human population; MtC, total meat consumption; C/MtC, domestic pork consumption/total meat consumption.

USDA [6–8]; The United Nations [9]; The World Bank [10].

